# Hierarchical Censored Bayesian Analysis of Visual Field Progression

**DOI:** 10.1167/tvst.10.12.4

**Published:** 2021-10-05

**Authors:** Giovanni Montesano, David F. Garway-Heath, Giovanni Ometto, David P. Crabb

**Affiliations:** 1 City, University of London, Optometry and Visual Sciences, London, UK; 2 NIHR Biomedical Research Centre, Moorfields Eye Hospital NHS Foundation Trust and UCL Institute of Ophthalmology, London, UK

**Keywords:** glaucoma, visual field, perimetry, hierarchical model, Bayesian

## Abstract

**Purpose:**

To develop a Bayesian model (BM) for visual field (VF) progression accounting for the hierarchical, censored and heteroskedastic nature of the data.

**Methods:**

Three versions of a hierarchical BM were developed: a simple linear (Hi-linear); censored at 0 dB (Hi-censored); heteroskedastic censored (Hi-HSK). For the latter, we modeled the test variability according to VF sensitivity using a large test-retest cohort (1396 VFs, 146 eyes with glaucoma). We analyzed a large cohort of 44,371 VF tests from 3352 eyes from five glaucoma clinics. We quantified the bias in the estimated rate-of-progression, the detection of progression (Hit-rate [HR]), the median time-to-progression and the prediction error of future observations (mean absolute error [MAE]). HR and time-to-progression were compared at matched false-positive-rate (FPR), quantified using permutations of a separate test-retest cohort (360 tests, 30 eyes with glaucoma). BMs were compared to simple linear regression and Permutation-Analyses-of Pointwise-Linear-Regression. Differences in time-to-progression were tested using survival analysis.

**Results:**

Censored models showed the smallest bias in the rate-of-progression. The three BMs performed very similarly in terms of HR and time-to-progression and always better than the other methods. The average reduction in time-to-progression was 37% with the BMs (*P* < 0.001) at 5% FPR. MAE for prediction was very similar among methods.

**Conclusions:**

Bayesian hierarchical models improved the detection of VF progression. Accounting for censoring improves the precision of the estimates, but minimal effect is provided by accounting for heteroskedasticity.

**Translational Relevance:**

These results are relevant for quantification of VF progression in practice and for clinical trials.

## Introduction

Glaucoma is a progressive optic neuropathy causing deterioration of the visual field (VF) as a consequence of the loss of retinal ganglion cells (RGCs) and their axons. VF testing is a staple of glaucoma care and is used both to diagnose glaucoma and monitor its progression. In most glaucoma clinics, standard automated perimetry is repeated at successive visits to assess progression of VF damage both for the whole field (global metrics) and at individual locations (pointwise analysis). VF damage in glaucoma usually occurs according to specific spatial patterns that reflect the organization of RGC axon bundles within the retina.^1^^–^^3^

Analysis of progression is, however, made difficult by complex features in VF data that can compromise their effective use in glaucoma care. A commonly used method to assess progression is ordinary least squares (OLS) regression, either on global or pointwise data. Assumptions for such a method are, however, often violated. For example, the variability of measured sensitivity is known to increase with the level of damage (heteroskedasticity), likely a consequence of the changes in response profile at damaged locations.^4^^,^^5^ Moreover, VF sensitivity is measured over a limited range. On one of the most commonly used devices, the Humphrey field analyzer (HFA; Zeiss Meditec, Dublin, CA, USA), the scale ranges from 50 dB to 0 dB. This scale is inversely related to the brightness of the stimulus, with 0 dB being the brightest. Such a lower bound on the measurement is completely arbitrary, and different cutoff values have been proposed.^6^^,^^7^ Nevertheless, a limited measurement scale is bound to produce censored data. However, values censored at 0 dB are often considered to be actual 0 dB for the scope of analysis. This can introduce positive biases in the measured progression rate, especially in VFs with more advanced damage, in which a trail of 0 dB values can arise in locations progressing beyond the measurement limits.^8^^,^^9^ Finally, it is often difficult to efficiently combine pointwise measurements to obtain a combined, easily interpretable, progression score for the whole VF without losing the rich information from individual locations.

A plethora of analysis methods have been proposed to deal with different aspects of VF data. Heteroskedasticity has often been neglected, given the complex nature of the relationship between response variability and sensitivity, providing variable amounts of improvement when incorporated in the analysis.^10^^,^^11^ Pointwise data have been combined using both scoring systems^10^^,^^12^^,^^13^ and multilevel models,^14^^,^^15^ almost invariably improving modeling performance. Data censoring has been dealt with only occasionally. Other workarounds have been proposed to deal with the 0 dB limits, such as asymptotic modeling through exponential decay,^13^^,^^16^^,^^17^ which, however, do not reflect the underlying nature of the data.

The scope of our work was to provide a comprehensive model that accounts for the key features of VF data based on our best knowledge of the underlying psychophysics of the test and the nature of sensitivity measurements. We also wanted a model whose parameters have a direct and meaningful interpretation for the clinician. To this aim, we propose a Bayesian implementation of a multilevel model for censored regression with heteroskedastic response. Similarly to previous Bayesian models,^10^^,^^15^ our method also accounts for clustering of locations within the VF according to the anatomy of RGC axon bundles.^1^ We measure the incremental improvement provided by censoring and heteroskedasticity by comparing different implementations of the model in terms of bias in the estimated progression rate, prediction error and clinical detection of progression using a large longitudinal cohort of 3352 eyes (44,371 VF tests). All these models are then benchmarked against other commonly available methods to assess VF progression.

## Methods

Our methodology has several connecting components. First, we describe the longitudinal VF data extracted from clinics and two independent test-retest data sets. Next, we outline our modeling of response variability, using one of the test-retest data sets. Then, we describe our new progression models along with the other models to be used as comparators. Finally, we outline how the models are tested and compared, using the clinical dataset to quantify detected progression and the second test-retest dataset to assess specificity.

### Datasets

#### Clinical Dataset

VF data were extracted from an EMR (Medisoft; Medisoft Ltd., Leeds, UK) from five regionally different National Health Service Hospital Trust glaucoma clinics in England in November 2015 as described elsewhere.^18^^–^^20^ In short, all patient data were anonymized at the point of data extraction and subsequently transferred to a single secure database held at City, University of London. Subsequent analyses of the data were approved by a research ethics committee of City, University of London. The study adhered to the Declaration of Helsinki and the General Data Protection Regulation of the European Union. All VFs were recorded on the HFA using a Goldmann size III stimulus with a 24-2 test pattern and the Swedish Interactive Testing Algorithms (SITA Standard or SITA Fast). The aggregated database contained 576,615 VFs from 71,361 people recorded between April 2000 and March 2015. We excluded any eye for which the EMR contained ocular surgery other than cataract removal during the follow-up period. We also excluded VFs with a percentage of false-positive errors ≥15%. No exclusion criteria were applied based on fixation losses or false-negative errors. For this study, we selected all patients with at least 10 VFs recorded over at least four years in one or both eyes and a mean deviation worse than −2 dB in at least two (not necessarily consecutive) VFs^21^^–^^23^ in the same eye. It seems likely that subjects with this level of damage and frequency of VF testing were either strong glaucoma suspects or persons with glaucomatous optic neuropathy. Finally, only one eye from each patient was selected, at random if both were eligible. The final selection included 44,371 VFs from 3352 eyes. Demographic details are reported in Table 1.

**Table 1.** Demographic Information for the Three Datasets Used in This Study, Reported as Median [Interquartile Range]

	Clinical Dataset	RAPID Dataset	HALIFAX Dataset
Age (years)	68 [60, 75]	70 [64, 76]	69 [64, 70]
BCVA (logMAR)	0 [−0.1, 0.2]	0 [−0.08, 0.18]	—
SE (D)	—	0 [−1.35, 0.88]	—
IOP (mm Hg)	16 [14, 18]	14 [12, 16]	—
Average MD (dB)	−6.44 [−11.06, −4.07]	−3.29 [−7.76, −1.24]	−2.57 [−4.36, −1.45]
Average PSD (dB)	5.68 [3.27, 9.06]	4.26 [2.16, 9.6]	3.11 [1.98, 5.39]

Average, patient-average calculations; BCVA, best corrected visual acuity; SE, spherical equivalent; D, diopters; IOP, intraocular pressure; MD, mean deviation; PSD, pattern standard deviation.

#### Test-Retest Datasets

Two independent test-retest datasets were used in this study. The first was the RAPID dataset.^24^^,^^25^ This dataset was used to obtain a model linking pointwise response variability with sensitivity (described in the next paragraph). Data were collected from stable eyes with primary open angle glaucoma at Moorfields Eye Hospital (reference 13/NS/0132) upon written informed consent. The data collection was in accordance with the Declaration of Helsinki. The final dataset used for this analysis was composed of 1396 test repeats performed in 146 eyes of 75 subjects. The number of test repeats per eye was 10 [7, 10] (median [95% quantiles]), with a minimum of 3 for inclusion. All tests were performed with a HFA 24-2 grid, SITA Standard strategy and a G-III stimulus size over an average time period of nine (maximum 13) weeks. The second was the HALIFAX dataset,^26^ as provided in *visualFields* package^27^ for R (R Foundation for Statistical Computing, Vienna, Austria). This dataset is composed of 12 VF test repeats from 30 eyes of patients with glaucoma and was used to create stable series through permutations to quantify the false-positive discovery rate (FPR = 1 – specificity, see section on model testing). The tests were performed with a HFA 24-2 grid, SITA Standard strategy and a G-III stimulus size over a time period of 12 weeks. Demographic characteristics for both datasets are reported in Table 1.

### Modeling of Response Variability

Variability was modeled as a function of sensitivity using the RAPID test-retest dataset (see previous paragraph). As previously mentioned, perimetric sensitivity values are censored at 0 dB. This means that thresholds lower than this limit are recorded as 0 dB. This can affect the observed test-retest distribution for a given location when the true sensitivity is close to this lower limit. To minimize this issue, we assumed that the test-retest distribution would ideally be symmetric around the true sensitivity value if the data were not censored. This is reasonable because perimetric strategies are designed to estimate the 50% threshold of the psychometric function.^28^ Then, for each location, we defined the median of the test-retest values as the best available estimate (BAE) of the true sensitivity for a given location. This gives an estimate of the central value of the test-retest of the distribution that is not affected by censoring (as opposed to the mean). When the median was 0 dB, we assumed that the BAE for the underlying threshold was not available and the location was removed from the analysis (5.1% of the tested locations).

**Figure 1.**
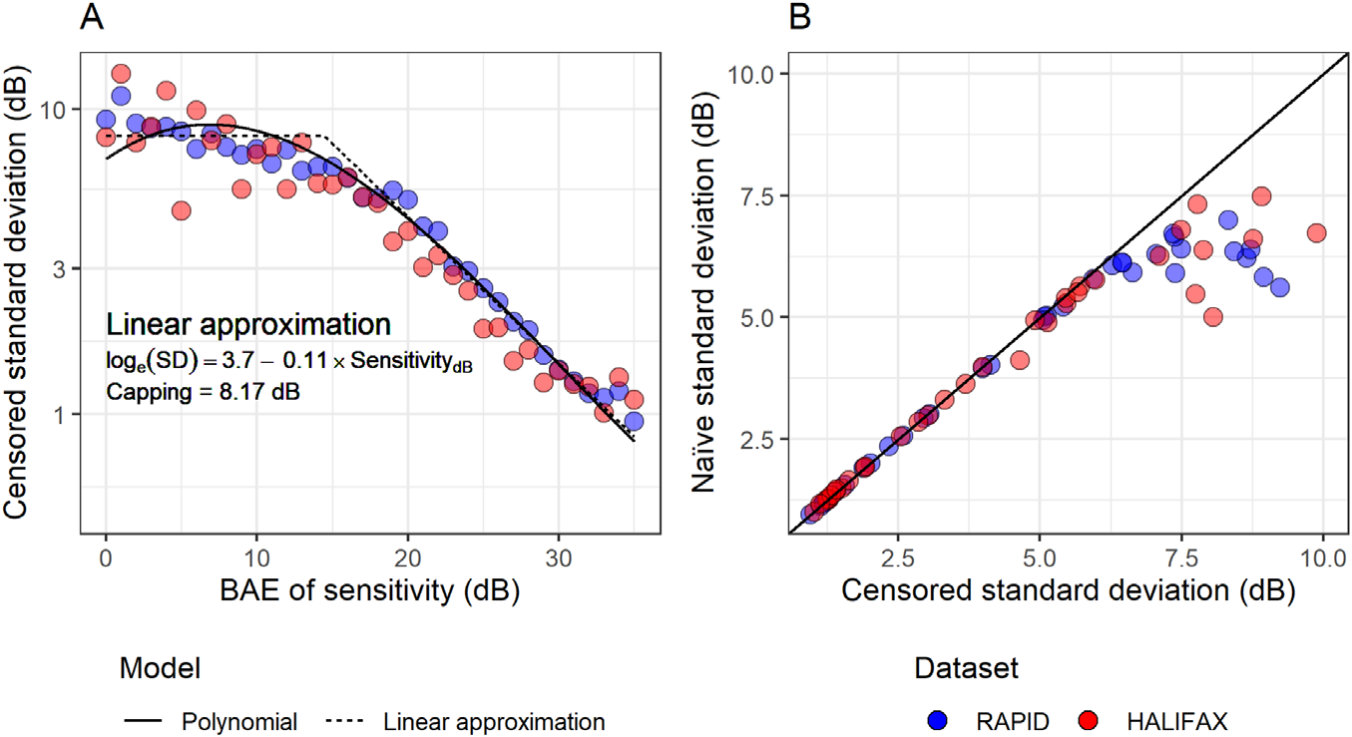
Panel A shows how the censored standard deviation for test-retest variability changes at different sensitivity levels and the corresponding predictions from the polynomial model and the bi-linear approximation. Panel B shows the comparison between naïve and censored standard deviation. Data-points are reported for both test-retest datasets but the models were only based on the RAPID dataset.

A censored heteroskedastic model, with a Gaussian error distribution, was then fitted via maximum likelihood using the package *crch* for R.^29^^,^^30^ The model allows one regression equation for the mean and one for the log(standard deviation [SD]). The mean was not modeled because the fitting was performed by including an offset term for each observation equal to the corresponding BAE of the true sensitivity. The log(SD) was modeled as a function of sensitivity. A good fitting was obtained with a third-degree polynomial. The coefficients for the polynomial model are reported in Appendix. However, for simplicity and speed of calculation in the implementation of our Bayesian model, we adopted an approximation where the relationship between log(SD) and sensitivity was linear in log-scale, with a maximum capping value (bilinear with one slope fixed at zero). This strategy has been previously adopted when implementing similar models of variability for simulation of perimetric responses.^31^ To estimate the capping value, the model for the log(SD) was a broken stick linear relationship where the break point was varied until the slope of the relationship for sensitivities lower than the break-point was null (i.e., no change). The coefficients for the linear approximation are reported in Figure 1. This relationship is in good agreement with the linear relationship described by Henson et al.^4^ obtained through direct measurements of the psychometric function. Figure 1A allows a direct comparison of the polynomial and approximated model of variability. For reference, we performed the calculations using the same methodology but modeling each (rounded) sensitivity value as a discrete factor level, so that a value of SD was calculated independently for each sensitivity value. This can then be compared with the naïve calculation of SD, which shows a clear bias at lower sensitivity, where the naïve calculation underestimates variability because of the censored values (Fig. 1B).

### Progression Models

For fairness of comparison, all methods were based on sensitivity values or their summary measurements, such as the mean sensitivity (MS). This was necessary for the correct modeling of censoring and heteroskedasticity at the pointwise level in the Bayesian models. These metrics, unlike the total deviation or the mean deviation, are also obviously affected by normal decline in visual function caused by aging. In the Discussion, we explain why this does not detract from the validity of the approach and how the effect of ageing could be accounted for in the calculations.

#### Proposed Mixed-Effect Hierarchical Bayesian Models

All models were developed in JAGS (Just Another Gibbs Sampler^32^) using *rjags* package for R.^33^ A separate model was fitted for each eye. We developed three versions of the hierarchical model. In its simplest form, the model was a linear regression of pointwise values over time (Hi-linear). The fixed effects were the global intercept and slope for the change of sensitivity over time. Hierarchical random effects on both intercepts and slopes were then added to model the change over time for different VF clusters (according to the map described by Garway-Heath et al.^1^) and for each location within a cluster. The prior distributions for the fixed effects were noninformative normal. The mean of these prior distributions and the starting points for the Markov Chain Monte Carlo (MCMC) algorithm were obtained from the fixed effect estimates of the same model fitted through ML (using the package *lme4* for R^34^). The random effects for the intercepts and slopes were modeled as bivariate noninformative normal distributions with zero mean. JAGS was then used to run the MCMC algorithm until convergence was achieved (Gelman-Rubin^35^ diagnostic ≤ 1.2 on two parallel chains) to numerically estimate the posterior distribution of the parameters. More details on the fitting procedure can be found in Appendix.

**Figure 2.**
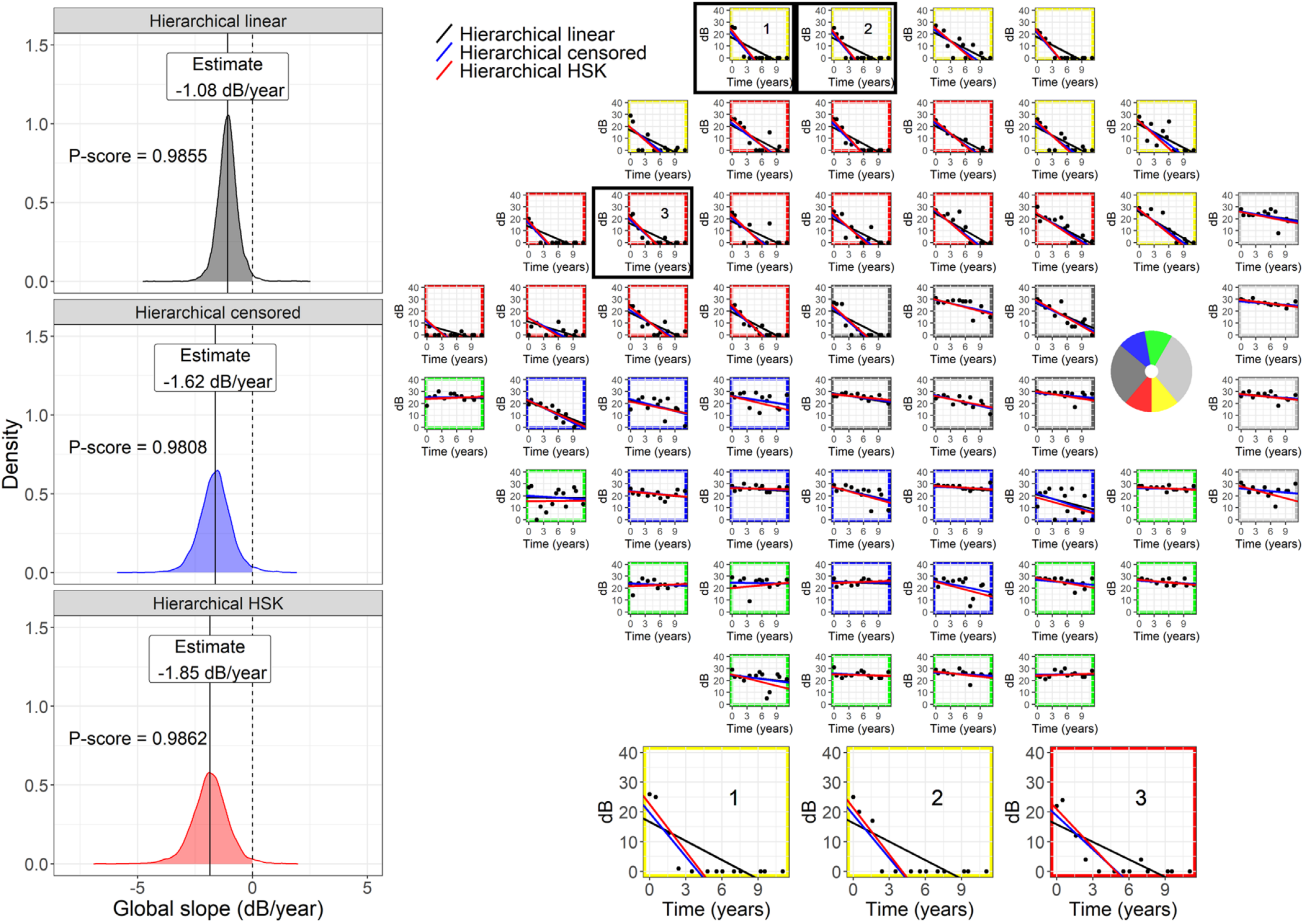
The panel on the right shows an example of a visual field series, fitted with the different hierarchical models. In the superior hemifield, it is evident how the censored models are less affected by the floor effect. The borders of the subplots are color-coded to indicate the cluster corresponding to different optic nerve head sectors (circular schematic in the blind-spot locations). The panels on the left show the posterior distribution for the global slope from the for the same field series, with the corresponding P-score, equivalent to the shaded area under the curve. The censored models produce less positively biased distributions, but with larger variance, reflecting the fact that censored sensitivity values only provide partial information. Hi = Hierarchical; HSK = Heteroskedastic.

Similarly to Betz-Stablein et al.,^15^ the posterior distribution for the global slope (fixed effect) was used to assess the global progression of the VF in each eye. The progression score (P-score) to assess progression was obtained by taking the value of the empirical cumulative distribution function of the posterior distribution of the global slope at 0 dB/year (no progression). In frequentist terms, the P-score is similar in interpretation to a one-sided *P* value for the slope: the closer to 1, the stronger the evidence for progression; a perfectly stable series would yield a P-score of 0.5; a P-score < 0.5 indicates a positive slope. An example is provided in Figure 2. One advantage of Bayesian inference over ML is that a posterior distribution can be estimated also for the random effects, making it possible to assess progression for individual clusters and locations using the same methodology. In other words, a P-score could be calculated in the same fashion using the posterior distributions of the random effects for the slope at the cluster and location level (see supplementary material for an example). Different cutoffs for the P-score were explored (see paragraph on model testing – clinical detection of progression). A second version of the model (Hi-censored) was identical to the one described, but the error distribution was a normal censored at 0 dB. This is similar in concept to a Tobit regression.^8^ However, the hierarchical mixed-model approach greatly overcomes the major limitation of unstable fitting results when only few uncensored values are available for specific locations. A third version of the model (Hi-HSK) also used a censored normal error distribution for sensitivity, but the SD of the error distribution was heteroskedastic and linked to the estimated sensitivity using the linear approximation reported in Appendix and previously described. Variability in this model was therefore imposed as a fixed relationship and not estimated, similarly to previous work.^10^

#### PoPLR

Permutation analyses of pointwise linear regression (PoPLR) was used as a term of comparison for progression detection.^12^ The method combines the *P* values for the slopes of pointwise linear regression (PLR) equations fitted to each location into a statistic S, using the Truncated Product Method. A customized null hypothesis distribution is then generated by calculating the S statistics on random permutations of the VF tests in the series and a *P* value for the S statistic is calculated. For our analysis, we used both the calculated S statistics and its *P* value. PoPLR only provides statistics for global progression. We used the implementation of the method provided by the *visualFields* package.^27^

#### Simple Linear Regression

Simple linear regression was also used as a term of comparison for our analysis. Separate equations were calculated for global progression (using the global MS), clusters (using the cluster MS), and pointwise sensitivity. The *P* value of the slopes from these equations was used to assess progression. These can be directly compared with the P-scores for the global slopes, the cluster slopes, and the location slopes from the Bayesian hierarchical models.

### Model Testing

#### Bias of the Estimates

This analysis was only performed for the Bayesian models. Our interest was to compare how different models were affected by observations censored at 0 dB. The expectation was that, as the amount of trailing 0 dB values in the series increased, the Hi-linear model would yield more positively biased estimates for the progression slopes compared to the implementations of the model with a censored error distribution. To test this, we selected a subset of VF series for which 0 dB values constituted <30% of the data points in the series for all locations (1486 eyes). This allowed us to have VF series relatively unaffected by censoring and that could be accurately described by a simple pointwise linear regression. Two additional artificial series were then created by shifting all the values in the original series down by 10 dB and 20 dB, generating artificial series with progressively lower sensitivity values. All values that fell below 0 dB when shifted were then censored. However, to obtain realistic data, we also increased the variability of the observations using the method described by Wu and Medeiros.^36^ Differently from the original method, the expected variability for different levels of sensitivities was not based on the empirical cumulative distribution function of the pointwise regression residuals but on a polynomial equation fitted to the HALIFAX test-retest database using the same methodology used to model variability in the RAPID dataset. This equation is also reported in Appendix. We finally compared, for each Bayesian model, the slopes estimated from the shifted series with the slopes obtained from the original series. Ideally, they should be equivalent, that is, the model least affected by censoring should give central estimates for the shifted series similar to the original series.

### Clinical Detection of Progression

This analysis was performed to compare the clinical performance of all the methods. For each method, we gradually changed the cutoff value of the progression metric to calculate a Hit-rate (HR) curves at different specificity levels. The HR was calculated on the 3352 VF series from the clinical database. The series were truncated at different lengths, from 4 VFs to 10 VFs (the minimum required for inclusion). Specificity was calculated by measuring the FP rate on stable series. The stable series were obtained as random permutations of those in the HALIFAX test-retest dataset. Each of the 3352 eyes in the clinical dataset was randomly paired to one of the 30 eyes in the HALIFAX dataset. Then, for each eye, the stable test-retest VF series was randomly permutated, generating 3352 stable series. The first 10 VFs of each permutation series were then retained and assigned the same time points as the real clinical series to replicate the calculations performed for the HR. The curves were calculated using the package *pROC* for R,^37^ up to a minimum specificity of 90%. Confidence intervals for the HR and the partial area under the HR curve (pAUC) were calculated with the bootstrap procedure implemented in the pROC package. Time to detect progression was quantified using survival curves for different specificity levels (97.5%, 95%, and 90%) using the package *survival* for R.^38^ Progression was assessed with a minimum of 4 VF tests on series truncated at a progressively increasing number of tests, up to a maximum of 10 tests. For each step in the series, all models were applied, and progression was detected on basis of the cutoff chosen for a given specificity level. The time to progression was then recorded as the earliest time from baseline where progression was detected. Formal comparisons between models were performed using a Cox proportional hazard model with a cluster term to account for the fact that progression was assessed on the same eye (VF series) with multiple methods. Note that survival analyses also account for the different number of actually observed progression events because eyes that did not progress within the observation window are considered as censored data. *P* values were corrected for multiple comparisons using the Bonferroni-Holm method.^39^ No statistical test was performed on the pAUCs because specificity for the HR curves was estimated using resampled permutated series that do not constitute an actual experimental sample.

### Prediction Errors

One concern with hierarchical models is the shrinkage of the random effect estimates (i.e., the cluster and location slopes) toward the grand mean. This might make the model “stiffer” and compromise prediction of future values. We therefore quantified the pointwise prediction error for each Bayesian method and compared it to the predictions from simple PLRs. All negative predicted values were assigned 0 dB. Predictions were performed in the first 10 VFs of each series and truncating the series at different lengths from 4 VFs to 9 VFs, predicting the rest. The mean absolute error (MAE) for all methods was also compared with the expected MAE for the test-retest series (from the HALIFAX dataset) and with predictions of no change from baseline, calculated as the average of the first two VF tests.

## Results

### Bias of the Estimates

**Figure 3.**
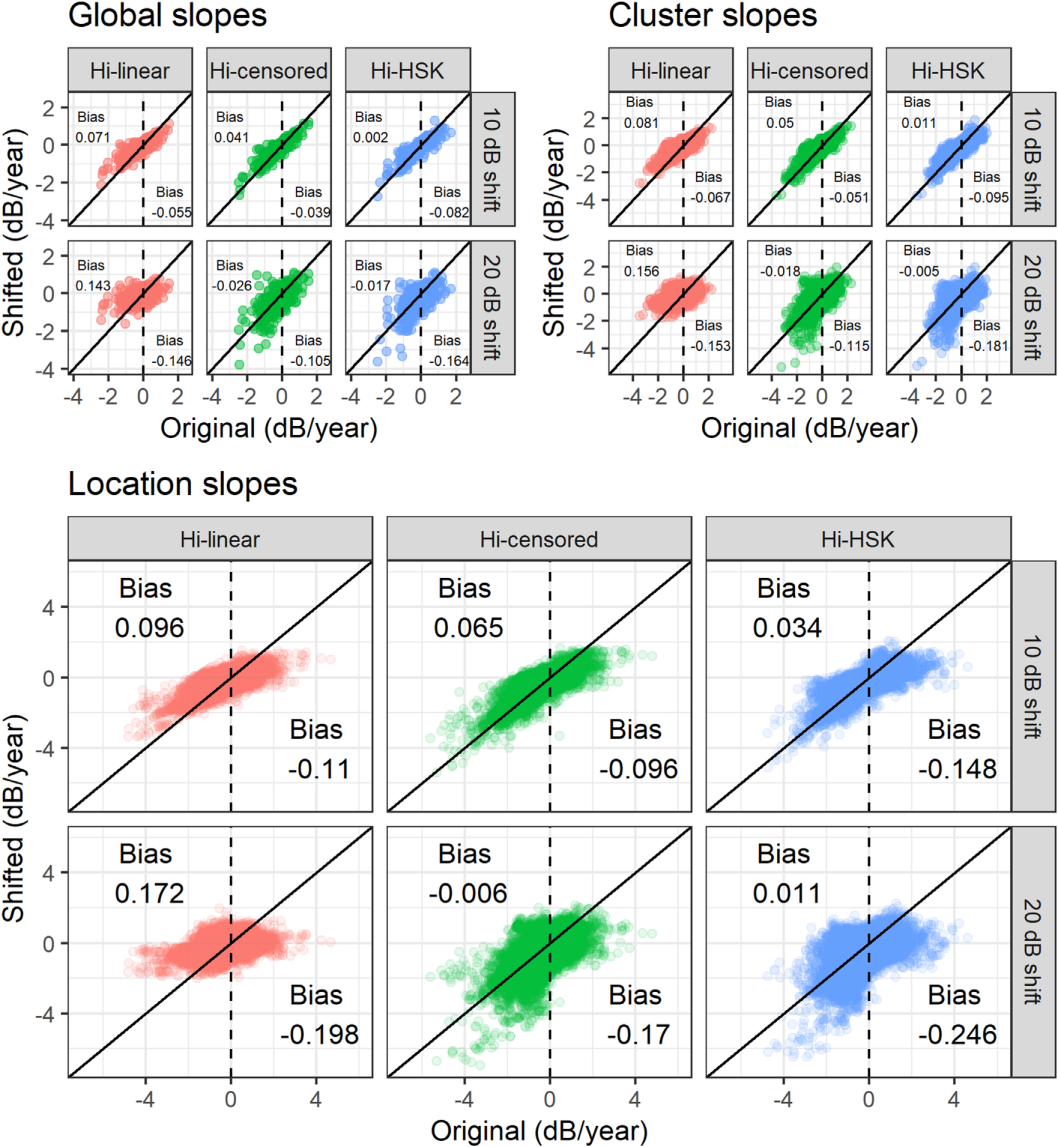
Comparison between the slopes estimated by the different models in the artificially shifted series and the original series. Note that the artificial series are shifted downward towards lower values. The censored models are less affected by a positive bias on the negative slopes compare to the Hi-linear model. The average bias for the negative and for the positive slopes is reported in the figure. Hi = Hierarchical; HSK = Heteroskedastic.

The agreement between progression slope estimates from the downward shifted artificial VF series and the original series is shown in Figure 3. As expected, the Hi-censored and the Hi-HSK models were the least affected by the 0 dB censoring, whereas a noticeable positive bias was evident for the negative slopes in the Hi-linear model. The average of bias is also reported in Figure 3. Of notice, the bias affected the cluster and location slopes, as well as the global slope. The effect of bias is also evident when plotting the distribution of slopes according to the intercept and when analyzing the distribution of slopes according to baseline VF damage (supplementary material).

### Clinical Detection of Progression

**Figure 4.**
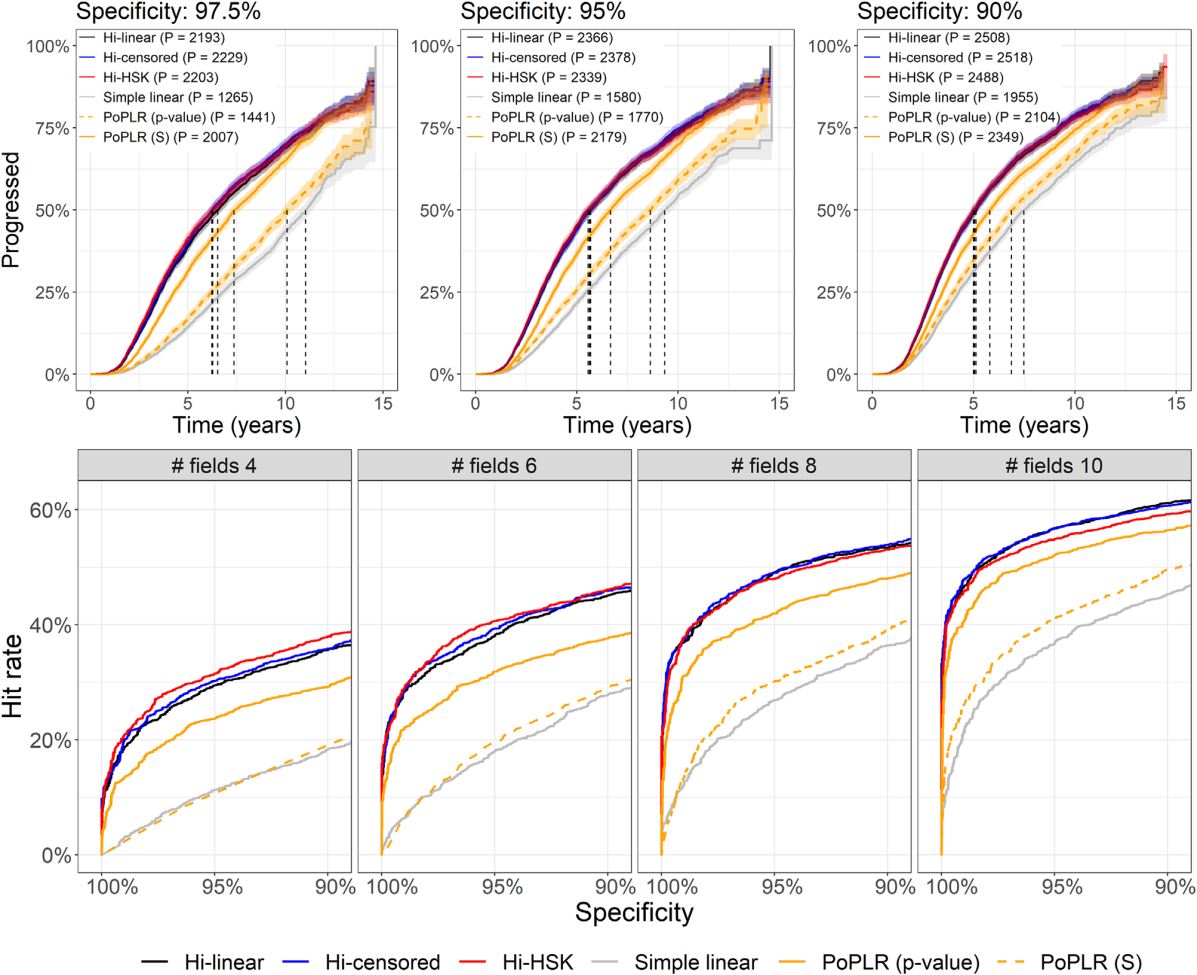
The top panels present the Kaplan-Meier curves for all tested methods at different specificity levels. P in the legends indicate the number of eyes that progressed with each method. The vertical dashed lines indicated the median time to progression (not color coded). The bottom panels show curves of the hit-rate at different specificity levels for all the progression methods tested with the series truncated at different lengths. Hi = Hierarchical; HSK = Heteroskedastic.

For global progression (Fig. 4) all Bayesian models performed very similarly and were vastly superior to both PoPLR statistics and simple linear regression for MS. Compared to the other hierarchical models, the Hi-HSK model was slightly superior for shorter series but performed slightly worse for longer series. Of note, the S statistics from PoPLR always performed better than the corresponding p-value statistics. PoPLR *P* value was significantly different from simple linear regression only with 8 and 10 VF tests (*P* < 0.001). Complete tables with partial areas under the curve and corresponding CIs are available as supplementary material.

The survival analysis and the formal comparisons mirrored the results seen for the HR curves (Fig. 4), in that all hierarchical models performed significantly better than the other methods (all *P* < 0.001) and PoPLR performed better than simple linear regression (*P* < 0.001). All hierarchical models performed very similarly and the only significant difference was detected at 97.5% specificity for the Hi-linear when compared to the Hi-censored (*P* < 0.001) and Hi-HSK (*P* = 0.0261) models. Table 2 reports the results of the survival analysis on global progression and the HR at three specificity levels.

**Table 2.** Median Time to Progression and Hit-Rate at Three Specificity Levels for Series Truncated at 10 Tests (the Minimum Required for Inclusion)

	Specificity		
Model	97.5%	95%	90%
Median time to progression (years [95% CIs]) (10 tests)			
Hi-linear	6.5 [6.2, 6.8]	5.7 [5.4, 5.9]	5.1 [4.9, 5.3]
Hi-censored	6.2 [6.0, 6.5]	5.6 [5.4, 5.9]	5.1 [4.9, 5.2]
Hi-HSK	6.3 [6.0, 6.6]	5.6 [5.3, 5.8]	5.0 [4.8, 5.2]
Simple linear	11 [10.7, 11.4]	9.3 [9.1, 9.7]	7.5 [7.2, 7.8]
PoPLR (p-value)	10.1 [9.8, 10.4]	8.6 [8.3, 8.9]	6.9 [6.7, 7.2]
PoPLR (S)	7.4 [7.1, 7.7]	6.7 [6.4, 6.9]	5.8 [5.6, 6]
Hit-rate (% [95% CIs]) (10 tests)			
Hi-linear	53 [51, 53]	57 [55, 57]	61 [59, 61]
Hi-censored	54 [51, 53]	57 [55, 57]	61 [59, 61]
Hi-HSK	52 [51, 52]	55 [54, 55]	59 [57, 59]
Simple linear	32 [29, 32]	37 [35, 37]	45 [43, 45]
PoPLR (p-value)	35 [33, 36]	41 [39, 41]	50 [47, 49]
PoPLR (S)	49 [47, 49]	52 [50, 52]	56 [55, 56]

Hi = Hierarchical; HSK = Heteroskedastic.

The 95% confidence intervals (CIs) were obtained from the Kaplan Meier estimator for the median time to progression and via bootstrap for the hit-rate.

**Figure 5.**
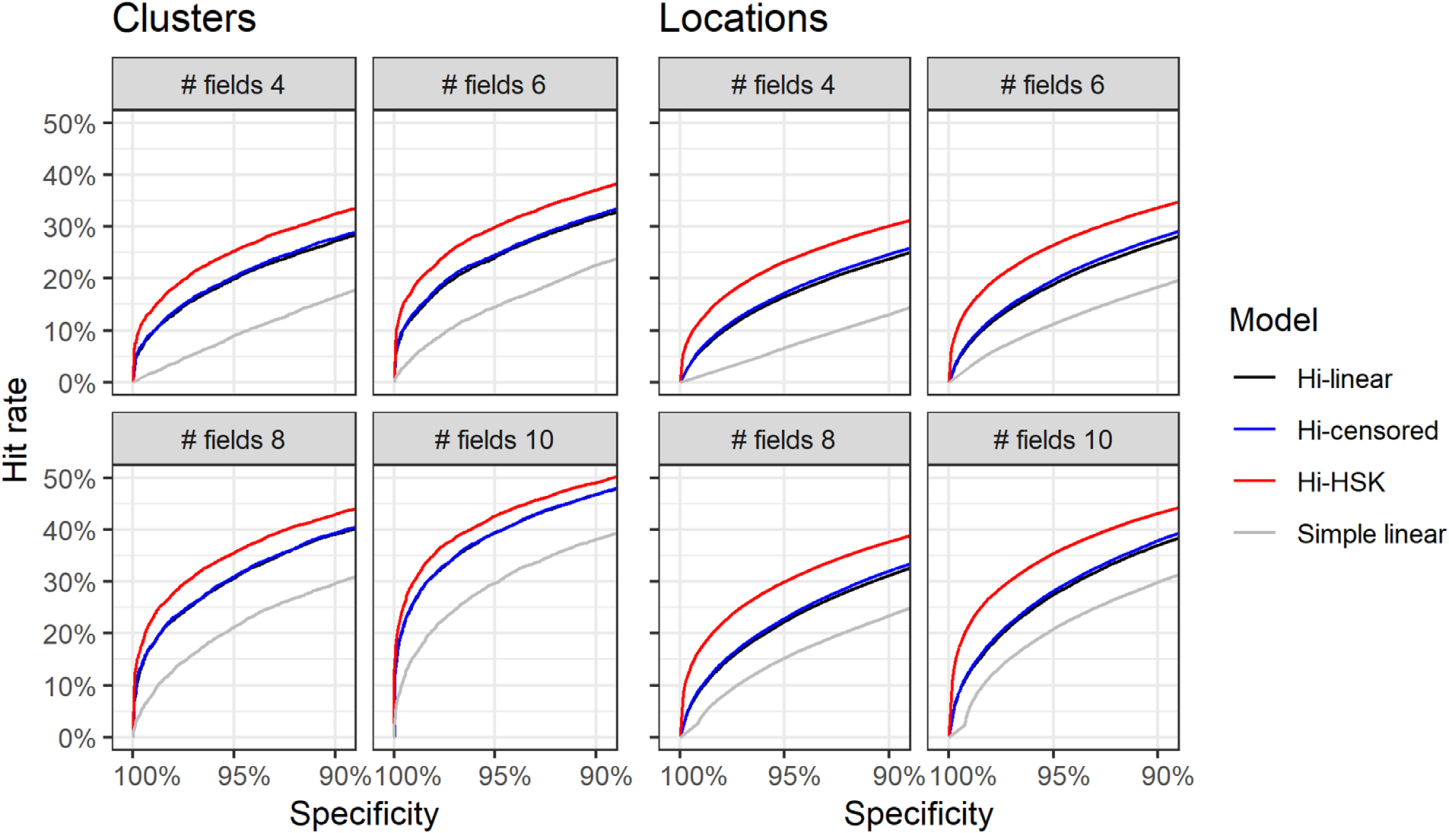
Curves of hit-rates at different specificities for individual visual field clusters and locations at different lengths of the series. Note that a “hit” is progression in any cluster, or location, and the percentages are calculated over the total number of clusters (N = 20,112) and locations (N = 174,304). Hi = Hierarchical; HSK = Heteroskedastic.

When applied to cluster and location progression (Fig. 5) the Bayesian hierarchical models performed much better than simple linear regression. The Hi-HSK model outperformed the other two hierarchical models for both clusters and locations. The differences between the Hi-censored and the Hi-linear models were minimal. Note that no formal testing was performed for these comparisons (see Methods), but pAUC values and the corresponding CIs are reported as supplementary material.

### Prediction Error

**Figure 6.**
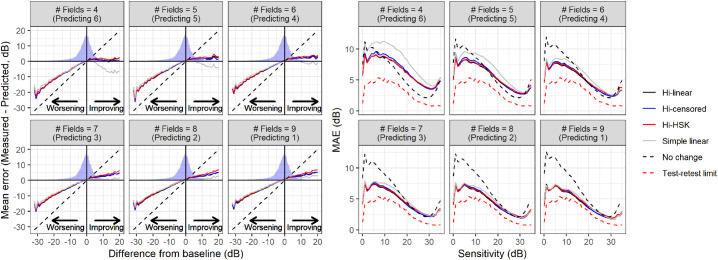
Average error for pointwise prediction stratified by difference from baseline (*left panel*) and mean absolute error stratified by baseline VF sensitivity (*right panel*) for the different hierarchical models and the pointwise linear regression. The density profile in the left panels represent the actual distribution of differences observed in the data. The diagonal dashed line represents the error resulting from predicting no change. Hi = Hierarchical; HSK = Heteroskedastic.

The MAE for predictions was generally better for the hierarchical models compared to simple PLR and prediction of no change (Fig. 6), with minimal differences among the three hierarchical models. Interestingly, at high sensitivities, the assumption of no change performed better than all models until 6 VFs were used to predict the remaining four. When stratified by difference from baseline, the signed prediction error was very similar between PLR and hierarchical models for worsening VFs but slightly better for the hierarchical models for improving fields (Fig. 6). Of note, large errors for extreme deviations from baseline persisted for all methods even when nine tests were used to predict the tenth. This is indicative of very large episodic deviations from linearity and might represent untruthful test results either in the VFs used to calculate the baseline or in those being predicted. The MAEs for different models are summarized in Table 3.

**Table 3.** Mean Absolute Error (MAE) for Prediction and the Corresponding Standard deviation (SD)

	MAE (SD)				
No. of Fields	Hi-Linear	Hi-Censored	Hi-HSK	Simple Linear	No Change
4	5.15 (6.59)	5.25 (7.38)	5.2 (7.25)	6.5 (9.54)	4.02 (4.85)
5	4.27 (5.12)	4.33 (6.10)	4.38 (6.16)	5.12 (6.44)	4.11 (4.94)
6	3.84 (4.65)	3.88 (5.34)	3.98 (5.47)	4.38 (5.44)	4.21 (5.04)
7	3.55 (4.16)	3.55 (4.26)	3.67 (4.39)	3.92 (4.67)	4.31 (5.15)
8	3.36 (3.98)	3.35 (4.06)	3.50 (4.21)	3.62 (4.34)	4.44 (5.29)
9	3.23 (3.85)	3.21 (3.91)	3.37 (4.07)	3.41 (4.08)	4.59 (5.46)

Hi = Hierarchical; HSK = Heteroskedastic.

MAE is in dB.

## Discussion

In this work, we evaluated three implementations of a hierarchical Bayesian model for VF progression. We separately investigated the effect of modeling the censored nature and the heteroskedastic behavior of VF data. The model for heteroskedasticity was based on a large test-retest dataset of glaucoma patients. The hierarchical models were then compared to more traditional approaches using a large clinical dataset with long VF series and permutations of stable VF series from an independent test-retest dataset. Our results highlight several important aspects of VF progression analysis, that are often overlooked.

In terms of clinical performance, the largest gains were provided by the multilevel approach combining different locations. Indeed, all three hierarchical models performed very similarly in detecting progression. This result is useful to interpret previous findings obtained with similar approaches. Zhu et al.^10^ also used a Bayesian approach to model VF progression. This method, called ANSWERS, was more focused on modeling heteroskedasticity (discussed later), but the parameter used to detect progression was a combination of the results obtained at different locations to calculate an estimated “probability of no progression.” In the evaluation of the method, the differential effect of modeling heteroskedasticity and combining locations was not quantified. Interestingly, when compared to simple linear regression, we obtained an improvement in performance very similar to that of ANSWERS for all the hierarchical models tested in our analysis, re-enforcing the idea that the strength of these methods comes from efficient combination of pointwise information. Importantly, we used the same dataset as Zhu et al.^10^ to obtain stable VF series to determine specificity, so our results can be easily compared. Of note, ANSWERS did not use a multilevel approach to obtain an estimate of the global progression slope but rather relied on a combination of pointwise regression models fitted individually (although linked through spatial correlations). This is similar in principle to the S statistics used by PoPLR, which also improved the clinical performance over simple linear regression in our dataset.

Other authors have successfully used a multilevel approach, similar to ours, to estimate a global progression slope.^14^^,^^15^ Betz-Stablein et al.^15^ in particular used the estimated posterior distribution on the global slope to assess progression. In their analysis, the hierarchical model did not show a large improvement over pointwise methods. However, they relied on clinical judgment to assess specificity in their calculations instead of permutations of stable series. Their results are therefore difficult to compare to ours. Nevertheless, modeling the global progression rate has additional advantages besides practical progression detection because it has a meaningful and direct interpretation for clinicians, and this is an additional strength of our approach.

Another novel aspect of our approach is the handling of the error distribution and response variability. Similarly to ANSWERS, we based our modeling on a test-retest dataset. In our case, however, the dataset used to model variability and the one used to generate permutated stable series were different, and this adds strength to our validation. Test-retest variability, especially at a pointwise level, is known to increase at lower sensitivities.^4^^,^^5^ This relationship is often explained with a change in the psychometric function^4^^,^^5^ at more damaged locations. Although this is certainly an incomplete characterization,^5^ sensitivity has been shown to be the best predictor of variability in glaucoma.^4^^,^^5^ Henson et al.^4^ modeled the psychometric function with a cumulative Gaussian function and reported the change of log(SD) with sensitivity, down to 10 dB. We adopted a similar modeling approach using a large test-retest perimetric dataset. The coefficients of our linear approximation were in good agreement with the results from Henson et al.^4^ (Appendix). Importantly, our approach was meant to model the expected variability around the predicted sensitivity, allowing us to rely on previous knowledge of the psychophysics of perimetric responses. In contrast, ANSWERS used test-retest data to model the variability of the observed response,^10^ effectively using the estimated variability as a weighting method for the observations. However, in our case, modeling heteroskedasticity only improved the results in the detection of progression for individual clusters and locations (Fig. 5) but did not improve the global performance.

One important feature of our hierarchical models is the handling of censored data. Different approaches have been proposed to address the 0 dB floor in perimetric measurements.^9^^,^^13^^,^^16^^,^^17^ However, few have addressed the actual censored nature of the data. This is important, because it implies that the actual sensitivity could extend beyond the measurements limits. This aspect is willingly neglected by methods adopting an asymptotic modeling of the floor effect,^13^^,^^16^^,^^17^ creating problems in the estimation and interpretation of the rate of progression. These methods are particularly problematic when considering that the censoring level is completely arbitrary and can be changed without any bearing on the measurements recorded above the chosen limit.^6^ This is not captured by asymptotic models, such as the exponential decay, in which the estimated rate of loss is tightly linked to the relative distance of the observations from the measurement limit. On the other hand, considering censored values as actual measurements of 0 dB sensitivity can introduce a positive bias in the estimated rate of loss with simple linear models. This is demonstrated by our first experiment on artificially shifted series (Fig. 3). It is important to notice that, at least in a dataset drawn from real clinics, such as the one used in this analysis, such a bias had little bearing on the ability of the models to predict future sensitivity, as demonstrated by our quantification of the MAE for predictions (Fig. 6), because a significant effect is only obtained when a large number of trailing 0 dB observations accumulate in a series (see example in Fig. 2). A similar result was reported by Bryan et al.^9^

Not accounting for censoring can, however, significantly affect the accurate estimation of the rate of loss in patients with advanced VF damage, because the floor is likely to affect many locations earlier in the VF series. Betz-Stablein et al.^15^ also accounted for the censored nature of the data. In contrast, ANSWERS used a transformation of 0 dB sensitivity because such a value would be undefined in a Weibull error distribution (the one used in their model).^10^ This approach allowed modeling of the observed test-retest variability, but would still be affected by a bias of trailing 0 dB values. However, despite reducing the estimation bias, accounting for censoring did not greatly improve the clinical performance (Fig. 4 and Fig. 5). This result could have multiple explanations, including the fact that our clinical dataset was mostly composed of people with early loss (see Table 1). This is representative of many glaucoma clinics, but in our case the number of people with early damage could have been inflated by our exclusion of eyes undergoing glaucoma surgery before 10 VFs could be collected. However, the most likely explanation is that models accounting for censoring correctly interpret censored observations as being less informative. This increases the uncertainty around the final estimate of the rate of progression, despite reducing the bias. Instead, noncensored models assume that complete information can be extracted from 0 dB measurements, leading to less uncertainty in the estimates. This is clearly illustrated by the example in Figure 2: both censored models provide more negative estimates of global progression, but the P-score is almost identical to that obtained with the Hi-linear model on account of the greater uncertainty. The comparison between the Hi-linear and the Hi-censored model is particularly useful, because they constitute two implementations of the same model that only differ for their handling of censored data. Other practical solutions could be applied to increase the dynamic range of VF testing itself at the lower end, for example, by using larger stimulus sizes for more advanced stages of damage.^40^

Finally, our approach differs from previous attempts in its modeling of spatial correlations within the VF. We opted for a full hierarchical approach, in which the VF clusters represented an intermediate level of the hierarchy. This has some important advantages, especially for interpretability, because the model can provide a rate of progression for each individual cluster. In fact, Bayesian computing allows inference on random effects, and this is useful to assess localized progression (Fig. 5). Moreover, the rate of progression for clusters can be compared to structural measurements on optic nerve sectors for multimodal evaluations.^25^^,^^41^ One drawback is that clusters are modeled as hard-edged groups instead of “smooth” correlations. Therefore, proximity of locations within the same cluster does not affect the correlation structure and adjacent locations in different clusters are modeled as completely independent. However, this also allowed us to avoid complex correlation structures in the model, greatly reducing the number of parameters and therefore improving efficiency, as opposed to previous similar attempts.^10^^,^^15^ Moreover, when compared to results from ANSWERS, our discrimination performance seems very close to those obtained with spatial enhancement in their model. For example, with five VF tests, they reported a 2.6-fold improvement in the HR compared to simple linear regression at 95% specificity.^10^ In our analysis, the improvement was 2.8-fold with the Hi-HSK model and 2.6 for the Hi-censored model. Note that results with 5 VFs are only reported here for comparison and are not part of our main analysis. Finally, we showed that our hierarchical structure with random effects retained enough flexibility so not to compromise its predictive ability (Fig. 6). These observations, together with the improved interpretability of the model, make our approach reasonable and, to some extent, preferable. Further flexibility could be introduced with customized structure-function clustering techniques.^3^^,^^42^

Other groups have proposed approximate Bayesian computation solutions to estimate VF progression, such as in the work by Murata et al.^43^^,^^44^ Their evaluation mostly focused on the improvement in prediction accuracy and did not explore the effect of their model on progression detection. Their results are difficult to compare to ours since they based their modeling on total deviation values. Interestingly, their MAE for prediction was similar but generally lower compared to ours. However, this was also the case for the simple pointwise linear regression, possibly indicative of a difference in the composition of the datasets used for validation, such as a larger proportion of stable patients. One notable difference is that we calculated the prediction MAE for all subsequent VFs and not just the last one in the series, as in Murata et al.^43^^,^^44^

### Limitations and Future Directions

Besides those pointed out in the previous section, our analysis has other limitations. Our clinical dataset recorded both actual 0 dB sensitivities and censored observations (denoted as <0 dB on HFA printouts) as 0. Therefore we had to assume that all 0 dB values indicated censored data. In future analyses, this distinction could be maintained to maximize the amount of information available to the model. However, this is unlikely to have greatly affected our results because the proportion of measured 0 dB is usually small compared to censored values. Another limitation is that the eyes included in our clinical dataset were not assigned a clear diagnostic label of glaucoma. However, it is reasonable to assume that people monitored in a glaucoma clinic with a minimum of 10 VFs over 4 years would be at least glaucoma suspects. Moreover, this limitation would only affect the amount of progressing eyes in the dataset but not the relative improvement observed between different methods.

In clinical care it is also common to select new baselines for VF progression analyses according to events such as a change in treatment or surgery. Therefore our analysis of 10 VFs as a continuous series might not capture this aspect of clinical practice. Evaluating the effect of selecting different baselines was beyond the scope of our model comparison. However, as for any other trend based analysis, this is certainly possible in practice. One final important aspect to consider is that our estimation of progression was based on sensitivity (rather than deviation) values and included both the effect of glaucoma and of normal ageing. Although this does not constitute a problem when comparing methods, it can be easily accounted for in clinical applications by changing the level of expected normal change. In our analysis, this was set to 0 dB/year (no change), but it could be modified to reflect the expected normal VF decline with ageing. However, no change is the appropriate choice when specificity is calculated on permutated test-retest series, because any effect of aging is minimal in these datasets, usually collected over a short period of time and completely eliminated by permutations.

A final obvious limitation is the long time taken to perform Bayesian computation. A detailed report on computation times for the different methods is provided as supplementary material. We also provide a comparison with the ML implementation of the Hi-linear model, which, despite being much faster and providing similar estimates, offered a worse clinical performance than its Bayesian counterpart. Finally, ML implementations of random effect models with censored error distribution are available,^45^ but they still rely on time-consuming numerical computations that offer little advantage over Bayesian implementations. Moreover, they do not allow for potential integration of prior knowledge, for example from structural data.^41^

One important application of this class of models is for the analysis of visual field outcomes in clinical trials for glaucoma treatments. Primary outcomes for these trials need to be sensitive enough to detect glaucoma progression in the relatively short time span of the trial. The hierarchical structure can be easily extended so that individual subjects constitute another level in the random effect structure while the fixed effects are used to model the differences in the rate of progression between, for example, two arms of the trial. Such an approach would overcome the limitations deriving from comparing survival curves of VF progression between the two arms, efficiently exploiting the information in the data to directly test the change in the rate of progression. In fact, Wu et al.^46^ showed how hierarchical models were more powerful than event based analyses, although they only analyzed the progression of mean deviation over time. A more complex hierarchical structure, making full use of pointwise data, has been used by our group in a previous analysis of trial data.^47^ That application was based on a ML procedure and did not account for censoring. Although this might be irrelevant when only people with early or no VF loss are recruited, the floor effect could bias the results where the focus is on more advanced visual field loss in people with later stage glaucoma.

## Supplementary Material

Supplement 1

## Appendix: Models for Variability

Table A1 reports the coefficients for the polynomial and linear approximation models of log(SD) of the response used in this analysis. The equations provided by Henson et al.^4^ are reported for reference. The polynomial fit used on the HALIFAX dataset was used to model the change in variability in the artificially shifted series to assess bias in the estimates of the slopes. Confidence Intervals are reported only for the linear approximation for comparison with Henson's results. The linear approximation was also recalculated with a break-point at 10 dB, the minimum sensitivity in Henson et al.^4^ Despite the small coefficient value, the cubic term in the polynomial fit significantly improved the goodness of fit (*P* < 0.001).

**Table A1.** Coefficients and Corresponding Confidence Intervals for Different Models

	Coefficients [95% Confidence Intervals]			
	Intercept	Sensitivity	Sensitivity^2^	Sensitivity^3^
Polynomial (RAPID)	1.93	0.08	−0.01	7 × 10^−5^
Polynomial (HALIFAX)	1.82	0.13	−0.01	0.0002
Linear approximation (optimized break-point)	3.70 [3.68, 3.72]	−0.111 [−0.112, −0.109]	—	—
Linear approximation (break-point at 10 dB)	3.45 [3.43, 3.47]	−0.101 [−0.103, −0.100]	—	—
Henson et al. (All subjects)	3.27 [2.98, 3.56]	−0.081 [−0.091, −0.071]	—	—
Henson et al. (Glaucoma)	3.62 [3.24, 4.00]	−0.098 [−0.112, −0.085]	—	—

Sensitivity is in dB. The linear approximation was capped at SD = 8.17 dB.

### Fitting of Bayesian Models

Two MCMC chains were run in parallel until convergence was achieved, defined as a Gelman-Rubin^35^ diagnostics < 1.2. We used 1000 samples for adaptation, 5000 burn-in samples and a minimum of 5000 samples to effectively sample the posterior distribution (more were added until convergence was achieved). Priors on the fixed effects (global intercept and slope) were non-informative normal distributions with a precision of 0.01 (Variance = 100) and the mean determined by the results of a simple ML fit on the same data (using the package *lme4*^34^). These were also the starting values for the MCMC.

The joint prior distribution for the random intercept and slope at each hierarchical level was a bivariate normal distribution with zero mean and a 2 × 2 precision matrix. The prior for the precision matrix was a Wishart distribution with a 2 × 2 inverse variance matrix with 0 off diagonal values and 2 degrees of freedom. Note that, in our implementation, the estimate for the intercept and slope parameters from the higher hierarchical level was cascaded down to the lower level as the actual mean of the random effect distribution.

The error distribution for the observations was a normal with zero mean (censored at 0 dB for the censored models). For the Hi-HSK model, the variance of the normal error distribution was determined based on the estimated sensitivity through the linear approximation equation presented in the previous section and was therefore not a fitted parameter. In preliminary evaluations of the model, this was deemed as the best method to achieve fast and stable convergence of the MCMCs. Tunable parameters with informative priors and more complex relationships, such as the third degree polynomial, generally performed worse in terms of clinical discrimination and stability of the estimates. When the residual variance of the observations was not imposed by a pre-determined sensitivity-variability relationship, it was modeled as a free precision parameter with a Gamma prior distribution with shape and rate parameters set to 0.001.
