# Supplementary material for: Hierarchical Censored Bayesian Analysis of Visual Field Progression
Source: Transl Vis Sci Technol. 2021 Oct 5;10(12):4. doi: 10.1167/tvst.10.12.4 (PMC8496414; doi:10.1167/tvst.10.12.4)
Supplement: Supplement 1 [file tvst-10-12-4_s001.pdf]

Supplementary to: Hierarchical censored Bayesian analysis of visual field progression

Intercept and slope distribution in the clinical dataset

Figure S1 shows a scatter-plot of the slopes and intercepts calculated with the different hierarchical models. The range of slopes is clearly limited for the Hi-linear model, especially for intercepts below 20 dB. This is particularly evident for clusters and locations.

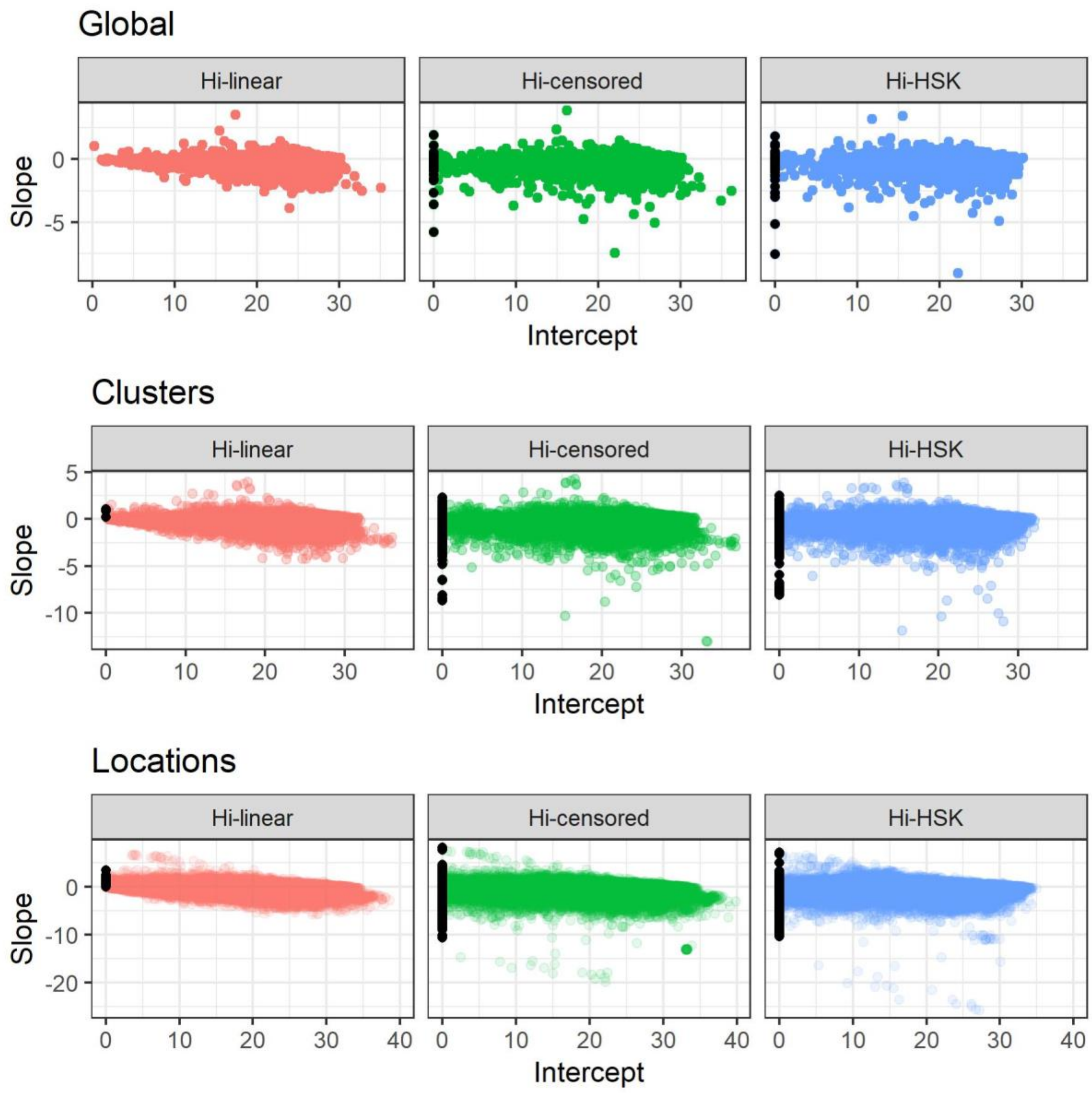

Figure S1

Table S1 reports descriptive statistics of the progression slopes calculated with the different models grouped by baseline Mean Deviation (MD, calculated as the average value of the first two visual field tests in each series.

	Slopes (dB/year), Median [Interquartile Range]		
	**MD > -6 dB**	**-6 dB < MD > -12 dB**	**MD < -12 dB**
**Hi-linear**	-0.21 [-0.47, -0.03]	-0.27 [-0.63, -0.06]	-0.23 [-0.5, -0.05]
**Hi-censored**	-0.22 [-0.48, -0.03]	-0.32 [-0.71, -0.06]	-0.39 [-0.78, -0.08]
**Hi-HSK**	-0.22 [-0.5, -0.05]	-0.35 [-0.73, -0.08]	-0.43 [-0.86, -0.09]

Table S1

Cluster progression - example

Figure S2 shows the posterior distributions and P-scores of the cluster slopes for the example in **Figure 2** in the main manuscript, using the Hi-Censored model.

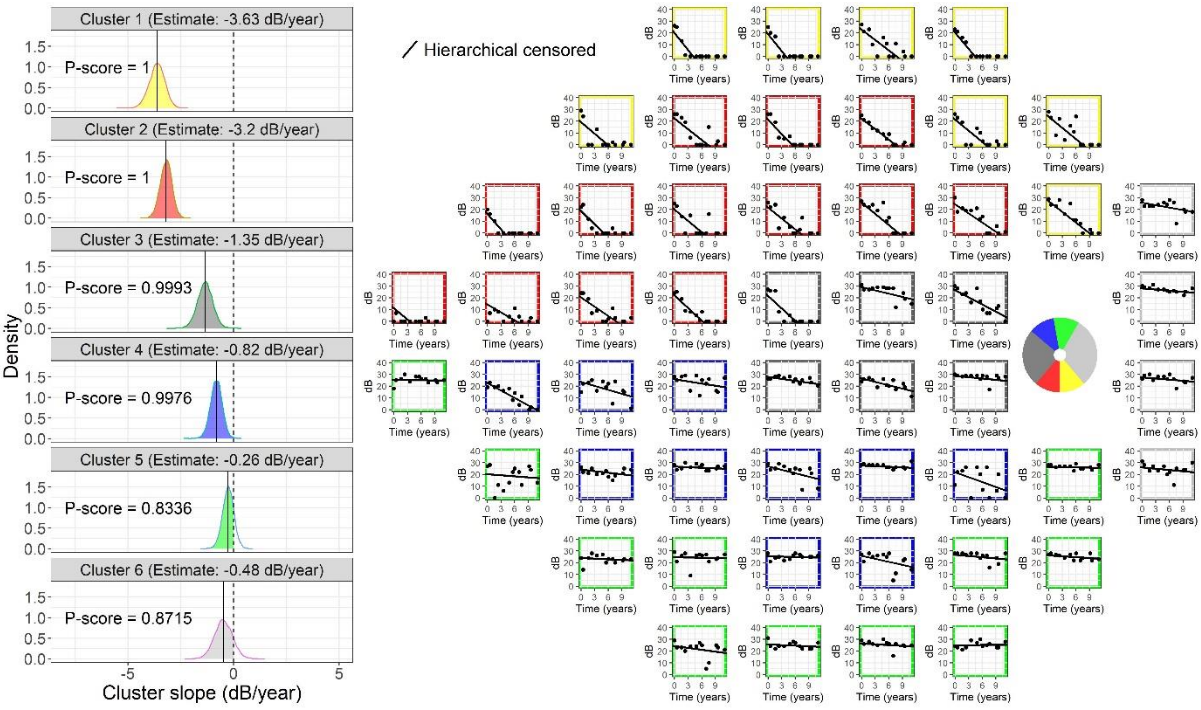

Figure S2

Partial areas under the hit-rate curves

Table S2 reports the partial areas under the hit-rates curve normalised over 0.1, the maximum achievable with specificity > 90%.

	partial Areas Under the Curve [95% Confidence Intervals]			
	**# fields 4**	**# fields 6**	**# fields 8**	**# fields 10**
		**Global**		
**Hi-linear**	0.28 [0.26, 0.29]	0.37 [0.35, 0.39]	0.47 [0.45, 0.49]	0.55 [0.53, 0.57]
**Hi-censored**	0.28 [0.27, 0.30]	0.38 [0.36, 0.40]	0.47 [0.45, 0.49]	0.55 [0.53, 0.57]
**Hi-HSK**	0.30 [0.28, 0.32]	0.38 [0.37, 0.40]	0.46 [0.44, 0.48]	0.53 [0.52, 0.55]
**Simple linear**	0.10 [0.09, 0.12]	0.17 [0.15, 0.19]	0.25 [0.23, 0.27]	0.35 [0.33, 0.37]
**PoPLR (S)**	0.22 [0.21, 0.24]	0.30 [0.28, 0.32]	0.40 [0.38, 0.42]	0.50 [0.48, 0.52]
**PoPLR (p-value)**	0.10 [0.09, 0.12]	0.18 [0.16, 0.20]	0.28 [0.26, 0.30]	0.39 [0.36, 0.41]
		**Clusters**		
**Hi-linear**	0.19 [0.18, 0.19]	0.23 [0.22, 0.24]	0.29 [0.28, 0.30]	0.37 [0.37, 0.38]
**Hi-censored**	0.19 [0.19, 0.20]	0.23 [0.23, 0.24]	0.29 [0.29, 0.30]	0.37 [0.37, 0.38]
**Hi-HSK**	0.24 [0.23, 0.25]	0.28 [0.28, 0.29]	0.34 [0.33, 0.35]	0.41 [0.40, 0.41]
**Simple linear**	0.09 [0.08, 0.09]	0.14 [0.13, 0.14]	0.20 [0.19, 0.21]	0.28 [0.27, 0.29]
		**Locations**		
**Hi-linear**	0.15 [0.15, 0.16]	0.18 [0.17, 0.18]	0.21 [0.21, 0.21]	0.26 [0.25, 0.26]
**Hi-censored**	0.16 [0.16, 0.16]	0.18 [0.18, 0.19]	0.21 [0.21, 0.22]	0.26 [0.26, 0.26]
**Hi-HSK**	0.22 [0.22, 0.22]	0.25 [0.25, 0.25]	0.28 [0.28, 0.28]	0.33 [0.33, 0.34]
**Simple linear**	0.07 [0.06, 0.07]	0.11 [0.10, 0.11]	0.14 [0.14, 0.14]	0.19 [0.19, 0.19]

Table S2

Computation times

All computations were performed on personal computer equipped with an Intel® Xenon® Silver 4110 CPU (2.10 GHz, 8 physical cores, 16 Logical processors) and 48 GB RAM. The computation of different eyes was performed in parallel but the calculation of each MCMC was not itself parallelised. The operating system was Microsoft Windows 10 Enterprise (64 bit). **Table S3** reports the computation time for each model.

	Computation time (seconds), Median [Interquartile Range]			
	**Hi-linear, ML**	**Hi-linear**	**Hi-censored**	**Hi-HSK**
**# Fields = 4**	0.21 [0.19, 0.22]	4.46 [4.31, 4.61]	4.53 [4.37, 4.71]	10.38 [10.21, 10.60]
**# Fields = 5**	0.22 [0.20, 0.24]	4.45 [4.28, 4.61]	4.55 [4.37, 4.74]	11.54 [11.35, 11.78]
**# Fields = 6**	0.24 [0.22, 0.26]	4.67 [4.53, 4.83]	4.81 [4.62, 5.00]	13.03 [12.81, 13.29]
**# Fields = 7**	0.26 [0.24, 0.28]	4.92 [4.78, 5.06]	5.05 [4.88, 5.27]	13.87 [13.63, 14.16]
**# Fields = 8**	0.28 [0.26, 0.30]	5.04 [4.90, 5.18]	5.19 [5.01, 5.42]	15.27 [14.97, 15.64]
**# Fields = 9**	0.30 [0.28, 0.32]	5.21 [5.06, 5.37]	5.39 [5.18, 5.66]	16.89 [16.54, 17.34]
**# Fields = 10**	0.32 [0.30, 0.35]	5.24 [5.10, 5.40]	5.45 [5.25, 5.74]	18.48 [18.11, 18.98]

Comparison with maximum likelihood estimates

Figure S2 shows the hit-rate curves for the Maximum Likelihood (ML) and Bayesian implementation of the Hi-linear model, compared to simple linear regression and PoPLR statistics. Despite being superior to simple linear regression, the performance of the ML implementation was much inferior

to its Bayesian counterpart. For longer series, it was also outperformed by the S statistics from PoPLR. However, the estimates for cluster and point-wise slopes were in good agreement between the two implementations (**Figure S3**).

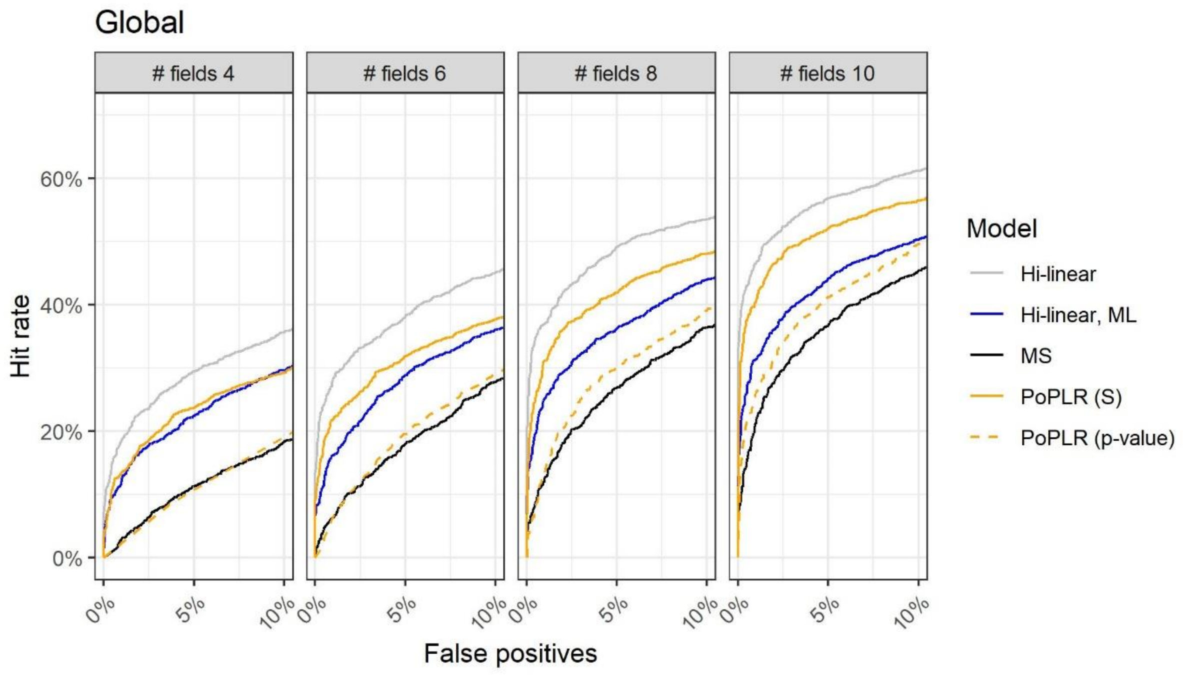

Figure S3

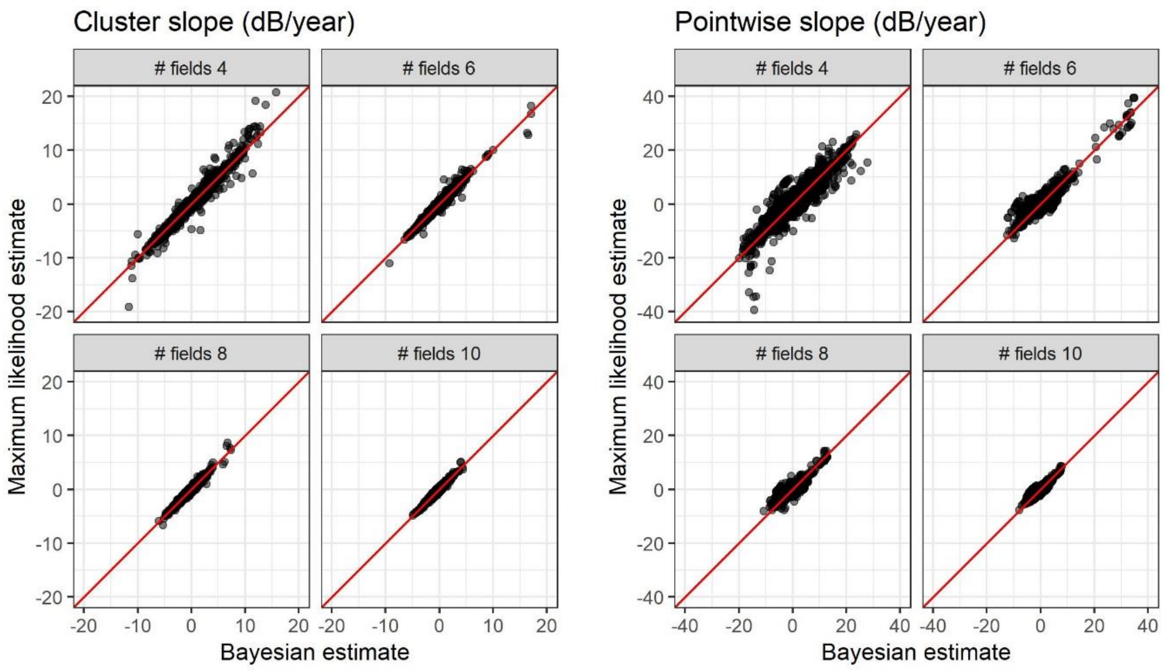

Figure S4
